# High-density Grid Use for Left Lateral Accessory Pathway

**DOI:** 10.19102/icrm.2021.120106S

**Published:** 2021-01-15

**Authors:** Chelsea Paskman, Andrea Hammond, John Harding, Robert Sangrigoli

**Affiliations:** ^1^Abbott, Chicago, IL, USA; ^2^Doylestown Hospital, Doylestown, PA, USA

**Keywords:** Advisor HD Grid, accessory pathway, AVRT, first burn termination, TactiCath sensor-enabled contact force ablation catheter

A 49-year-old woman with multiple documented episodes of narrow QRS complex tachycardia underwent electrophysiology study at our hospital. Baseline ventricular pacing demonstrated eccentric retrograde coronary sinus activation consistent with a left lateral accessory pathway **(Figure 1A)**. Orthodromic tachycardia using a left lateral accessory pathway was repeatedly induced.

Transseptal puncture allowed access to the left atrium and left ventricle, where high-density mapping was performed across the mitral valve anulus during ventricular pacing using the Advisor™ HD Grid Mapping Catheter, Sensor Enabled™ and the EnSite Precision™ electroanatomic mapping system to localize the concealed left lateral accessory pathway. The open-window mapping technique^1^ was used by collecting the absolute dV/dt bipolar electrogram from the high-density grid to help distinguish the mitral valve anulus, collecting both atrial and ventricular electrograms to delineate functional block located at the valve plane **(Figure 1B)**. At the location of the accessory pathway, bipolar fusion and pathway potentials recognized and annotated on the high-density grid were able to showcase the atrial insertion point of the accessory pathway **(Figures 1B and 1C**). Additionally, during ventricular pacing and, similarly, during orthodromic supraventricular tachycardia, EnSite Precision™ open-window propagation and, in particular, the SparkleMap™ display feature **(Figures 2A and 2B)** allowed for more precise localization of the atrial insertion of the bypass tract by dynamically displaying wavefront propagation superimposed on top of the local activation timing and voltage maps **(Videos 1 and 2)**.

Ablation was performed with the TactiCath™ Contact Force Ablation Catheter, Sensor Enabled™ at a power of 30 W and a minimal contact force of 10 g while the patient was in orthodromic supraventricular tachycardia. The tachycardia was terminated with a single lesion delivered to the mitral valve annulus at the atrial insertion site, subsequently eliminating the accessory pathway **(Figure 1E)**. A thorough waiting period and electrophysiology study showed no evidence of accessory pathway conduction and the patient has remained symptom-free during follow-up.

## Figures and Tables

**Figure 1:**
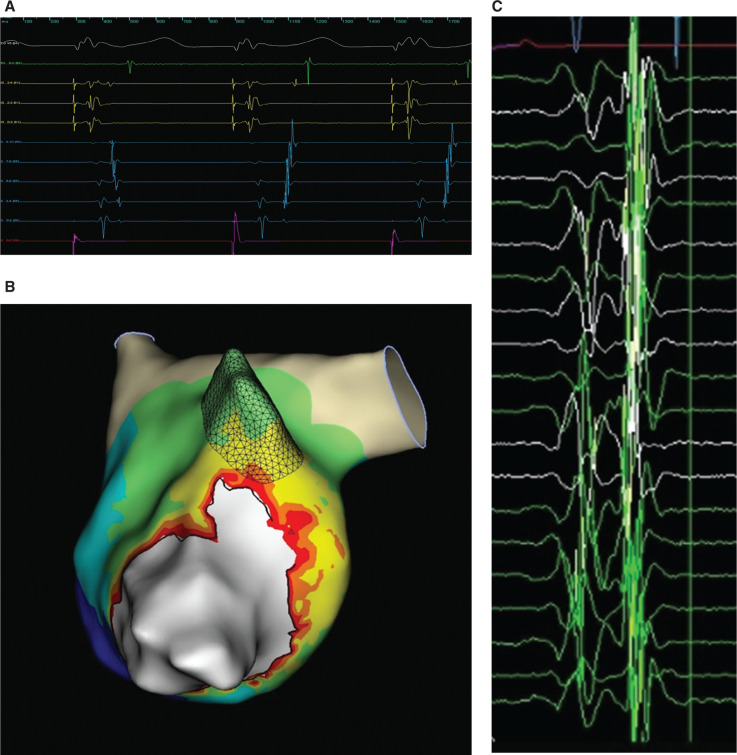
**A:** Ventricular pacing showcasing retrograde activation switching from atrioventricular nodal to accessory pathway conduction. **B:** EnSite Precision™ open window map of the left lateral accessory pathway using the Advisor™ HD Grid catheter. **C:** Advisor™ HD Grid electrogram at the left lateral pathway location.

**Figure 1:**
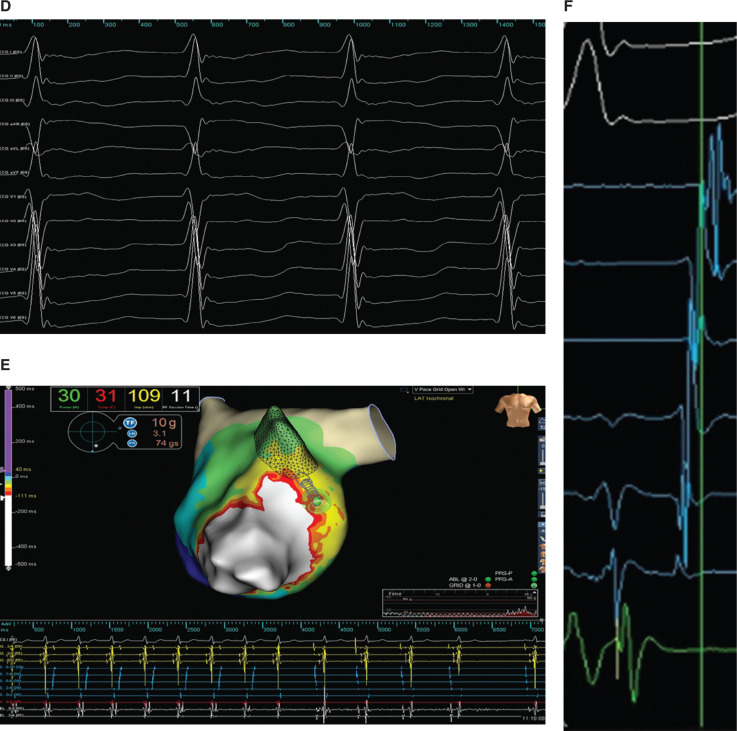
**D:** Clinical tachycardia 12-lead presentation. **E:** First burn termination during supraventricular tachycardia with the TactiCath™ contact force catheter. **F:** Ventriculoatrial fusion at the site of successful ablation on the pathway.

**Figure 2:**
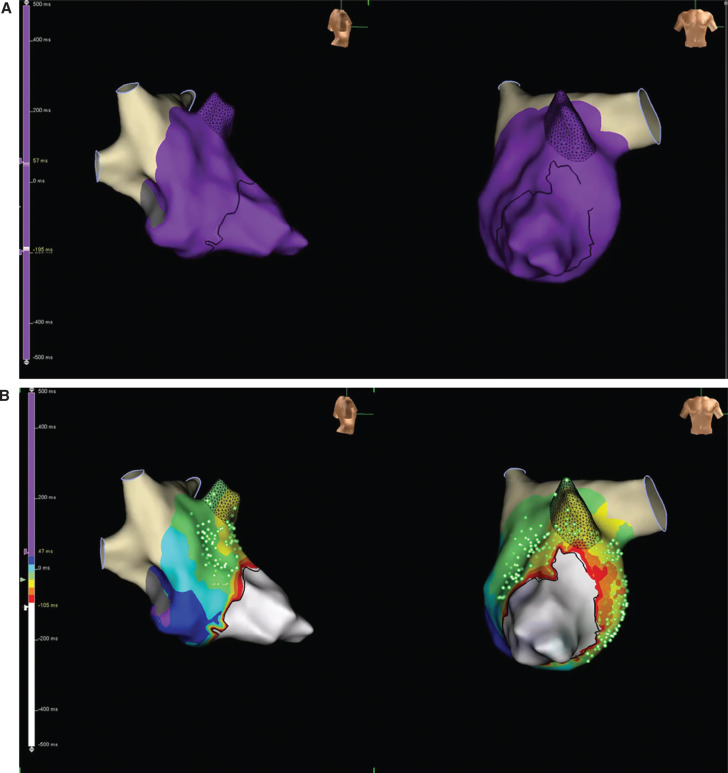
**A:** EnSite Precision™ open-window propagation video of the left lateral accessory pathway. **B:** EnSite Precision™ open-window map of the left lateral accessory pathway displayed with SparkleMap™.

**Video 1.** EnSite Precision™ open window propagation video of the left lateral accessory pathway.

**Video 2:** EnSite Precision™ open window map of the left lateral accessory pathway displayed with SparkleMap™.
